# Zoonotic Pathogens in *Ixodes scapularis*, Michigan

**DOI:** 10.3201/eid1307.070046

**Published:** 2007-07

**Authors:** Sarah A. Hamer, Pamela L. Roy, Graham J. Hickling, Edward D. Walker, Erik S. Foster, Christina C. Barber, Jean I. Tsao

**Affiliations:** * Michigan State University, East Lansing, Michigan, USA; † University of Tennessee, Knoxville, Tennessee, USA; ‡ Michigan Department of Community Health, East Lansing, Michigan, USA

**Keywords:** Ixodes scapularis, Lyme disease, human anaplasmosis, Borrelia burgdorferi, Anaplasma phagocytophilum, Babesia odocoilei, Michigan, letter

**To the Editor:** *Ixodes scapularis*, the black-legged tick, is the predominant vector of reportable human vectorborne disease in the United States. It transmits agents that cause Lyme borreliosis, human anaplasmosis, and human babesiosis. *I*. *scapularis*–borne disease is becoming more frequent as this tick expands its range from tick-endemic foci in the northeastern and upper midwestern United States.

Despite Michigan’s proximity to large tick-endemic areas (Wisconsin and Minnesota to the west and Indiana to the south), active and passive surveillance data indicated that the only populations of *I. scapularis* established in the state before 2002 were in Menominee County in the Upper Peninsula (*1**,**2*). However, wildlife sampling and tick dragging in 2002–2003 suggested that *I*. *scapularis* had begun to invade southwestern Michigan (*3*), with nearby populations in northwestern Indiana (*4*) as the putative source.

Because we suspected these invading ticks were bringing zoonotic pathogens into southwestern Michigan, we assessed pathogen prevalence within the state’s invading and endemic *I*. *scapularis* populations. Over a 1.5-week period in April–May 2006, we collected adult *I*. *scapularis* by drag sampling at 3 recently invaded sites in southwestern Michigan and 2 tick-endemic sites in Menominee County. We targeted adult *I*. *scapularis* in the spring because this life stage has had 2 chances of becoming infected and because the adult questing peak in Michigan is greater in spring than fall (*2**,**3*).

All collected ticks were bisected aseptically, and total DNA was extracted from half after overnight lysis (DNeasy Tissue Kit; QIAGEN, Valencia, CA, USA). We used 3 PCRs to assay for *Borrelia burgdorferi*, *B*. *lonestari*, and *B*. *miyamotoi* (*5*); *Anaplasma phagocytophilum* (*6*); and *Babesia* spp., including *Babesia microti* and *Babesia odocoilei* (*7*). *Borrelia*-positive and *Babesia*-positive amplicons were purified and sequenced for species identification.

Tick densities were highest overall at tick-endemic Menominee County sites; in southwestern Michigan, they were highest at those sites closest to the putative source of the Indiana invasion. We collected 28 adult and 1 nymphal *I*. *scapularis* and 2 adult *Dermacentor variabilis* from tick-endemic sites. Of the adult *I*. *scapularis*, 17 (60.7%) were positive for *B*. *burgdorferi*, 4 (14.3%) were positive for *A*. *phagocytophilum*, and 2 (7.1%) were positive for *Babesia odocoilei* (Table). We also collected 91 adult and 10 nymphal *I*. *scapularis* and 5 adult *D*. *variabilis* from newly invaded sites. Of the adult *I*. *scapularis*, 43 (47.3%) were positive for *B*. *burgdorferi*, 1 (1.1%) was positive for *A*. *phagocytophilum*, and 4 (4.4%) were positive for *Babesia odocoilei*. All 4 *Babesia odocoilei*–positive ticks were co-infected with *B*. *burgdorferi* (this rate of co-infection was significantly greater than random expectation; p = 0.046, by Fisher exact test).

**Table:** Prevalence of 3 pathogens in *Ixodes scapularis* ticks from 2 Michigan field sites, spring 2006*

Site	Life stage	No. *I. scapularis*	No. ticks infected or co-infected (%)				
			*Borrelia burgdorferi*	*Anaplasma phagocytophilum*	*Babesia odocoilei*	*B. burgdorferi* and *A. phagocytophilum*	*B. burgdorferi* and *B. odocoilei*
E-1	A	16	9 (56.3)	1 (6.3)	1 (6.3)	1 (6.3)	0
E-2	A	12	8 (66.7)	3 (25.0)	1 (8.3)	1 (8.3)	1 (8.3)
	N	1	1 (100.0)	0	0	0	0
I-1	A	4	2 (50.0)	0	0	0	0
	N	2	0	1 (50.0)	0	0	0
I-2	A	18	9 (50.0)	0	1 (5.6)	0	1 (5.6)
	N	8	2 (25.0)	1 (12.5)	0	1 (12.5)	0
I-3	A	69	32 (46.4)	1 (1.4)	3 (4.3)	0	3 (4.3)
All endemic sites	A	28	17 (60.7)	4 (14.3)	2 (7.1)	2† (7.1)	1† (3.6)
	N	1	1 (100)	0	0	0	0
All invaded sites	A	91	43 (47.3)	1 (1.1)	4 (4.4)	0	4‡ (4.4)
	N	10	2 (20.0)	2 (20.0)	0	1† (10.0)	0

*E, endemic site; A, adult: N, nymph; I, invaded site. †Nonsignificant level of co-infection; p = 0.378–0.640, by Fisher exact test. ‡Significant level of co-infection; p = 0.046, by Fisher exact test.

Within the tick-endemic area, comparison with prior survey data (*8*) indicated that the *B*. *burgdorferi* infection rate in adult ticks increased from 31.3% in 1992 to 60.7% in the present survey (p<0.001, by Fisher exact test). A similar increasing trend was evident in the invasion area, where prevalence increased from 37.0% in 2002–2003 (at a collection site 5 km south of our southernmost site; [*3*]) to 47.3% in 2006. This latter trend was only marginally statistically significant due to small sample size and the short period between surveys (p = 0.046, by 1-tailed Fisher exact test).

*B*. *burgdorferi* infection in *I*. *scapularis* has been reported in Michigan (*1**–**3**,**8*). To our knowledge, ours is the first report of *A*. *phagocytophilum* and *Babesia odocoilei* in ticks in Michigan; they are present in both the endemic and recently invaded populations. Similar infection rates for these pathogens have been reported in *I*. *scapularis* from Indiana (*9*). *B*. *burgdorferi* and *A*. *phagocytophilum* are human pathogens; *Babesia odocoilei*, an intraerythrocytic protozoan parasite maintained in transmission cycles in white-tailed deer, is not known to be pathogenic to humans (*7*). Several other *Borrelia* and *Babesia* species (i.e., *B*. *lonestari*, *B*. *miyamotoi*-like spirochetes, and *Babesia microti*) from US ticks were not detected in our sample. *I*. *scapularis* nymphs, which are epidemiologically important (*10*), were not the focus of our sampling. However, several were collected, including some infected with *B*. *burgdorferi*, *A*. *phagocytophilum*, or both (Table).

These data imply a risk for Lyme borreliosis and human anaplasmosis in areas endemic for and recently invaded by *I*. *scapularis*. For example, Lyme disease incidence in the tick-endemic zone has increased significantly over the past 10 years (from 0.33 to 1.53 cases per 10,000 persons during 1997–2006; *r^2^* = 0.56, p = 0.01). Incidence in the invasion zone has been much lower (mean 0.03 cases per 10,000 persons over same period) but appears to be increasing. Further increases in tick population size, infection, and co-infection can be expected as the invasion continues (*9*). Thus, medical practitioners in southwestern Michigan should be aware of the changing increasing risk for tickborne diseases and consider disease resulting from these pathogens during diagnosis.
